# Novel models for early prediction and prevention of acute respiratory distress syndrome in patients following hepatectomy: A clinical translational study based on 1,032 patients

**DOI:** 10.3389/fmed.2022.1025764

**Published:** 2023-01-09

**Authors:** Xiaoqiang Wang, Hongyan Zhang, Ruiqing Zong, Weifeng Yu, Feixiang Wu, Yiran Li

**Affiliations:** ^1^Department of Intensive Care Medicine, Eastern Hepatobiliary Surgery Hospital, The Third Affiliated Hospital of Naval Medical University, Shanghai, China; ^2^Department of Anesthesiology, Renji Hospital, Shanghai Jiao Tong University School of Medicine, Shanghai, China

**Keywords:** acute respiratory distress syndrome, liver cancer, hepatectomy, prediction model, organ failure, LASSO regression

## Abstract

**Background:**

Acute respiratory distress syndrome (ARDS) is a serious organ failure and postoperative complication. However, the incidence rate, early prediction and prevention of postoperative ARDS in patients undergoing hepatectomy remain unidentified.

**Methods:**

A total of 1,032 patients undergoing hepatectomy between 2019 and 2020, at the Eastern Hepatobiliary Surgery Hospital were included. Patients in 2019 and 2020 were used as the development and validation cohorts, respectively. The incidence rate of ARDS was assessed. A logistic regression model and a least absolute shrinkage and selection operator (LASSO) regression model were used for constructing ARDS prediction models.

**Results:**

The incidence of ARDS was 8.8% (43/490) in the development cohort and 5.7% (31/542) in the validation cohort. Operation time, postoperative aspartate aminotransferase (AST), and postoperative hemoglobin (Hb) were all critical predictors identified by the logistic regression model, with an area under the curve (AUC) of 0.804 in the development cohort and 0.752 in the validation cohort. Additionally, nine predictors were identified by the LASSO regression model, with an AUC of 0.848 in the development cohort and 0.786 in the validation cohort.

**Conclusion:**

We reported the incidence of ARDS in patients undergoing hepatectomy and developed two simple and practical prediction models for early predicting postoperative ARDS in patients undergoing hepatectomy. These tools may improve clinicians’ ability to early estimate the risk of postoperative ARDS and timely prevent its emergence.

## Introduction

The acute respiratory distress syndrome (ARDS) was first described in 1967, and specific diagnostic criteria were established in 1992 by an American-European consensus meeting (1, 2). ARDS is a common and serious complication that affects patients worldwide and often leads to a poor prognosis or even death (3). A prospective analysis of the ARDS incidence in the United States from 1999 to 2000 (4) estimated an annual incidence of 190,000 cases of ARDS in the United States, with a surprising hospital mortality rate of 38.5%. Another multicenter prospective study called LUNG-SAFE evaluated intensive care units (ICU) incidence and outcome of ARDS in 29,144 patients from 50 different counties (5). According to the findings, the prevalence of ARDS in ICU patients reached 10%, while it was identified in 23% of all patients receiving ventilation. In addition, the LUNG-SAFE study reported that the hospital mortality rate was 34.9% for patients with mild ARDS and 46.1% for those with severe ARDS. Furthermore, the global burden of ARDS is the highest in high-and upper-middle-income countries, with data indicating that the mean total hospitalization costs in Korea reached 12,336 United States dollars (USD) (6, 7). Therefore, the high morbidity and mortality of ARDS significantly reduce patients’ prognosis and place a heavy burden on global health.

There are many risk factors that contribute to ARDS development, such as pneumonia, non-pulmonary sepsis, major trauma, including surgical and accidental trauma, aspirations of gastric contents, and others (8, 9). In addition, other factors such as alcohol consumption, smoking, and hypoalbuminemia have all been linked to an increased risk of ARDS (10–12). Although great strides have been made in learning and treating ARDS over the last 50 years, effective preventive measures for postoperative ARDS still require further investigation (13, 14). Previous studies using administrative or perioperative data have established certain scoring models for predicting the risk of postoperative pneumonia, acute lung injury (ALI), or ARDS in patients (15–17). However, the majority of these studies built models based on either the general population or multiple types of surgery, and many of the predictors included in the models were inapplicable to patients undergoing specific type of surgery (16, 18, 19).

According to the most recent data, liver cancer is one of the most common cancers and the third leading cause of death among all cancers (20). Hepatectomy is a major surgical procedure that is commonly used for treating liver tumors (21). Despite improvements in hepatectomy safety over decades, the frequency of postoperative complications remains significant, ranging from 4.09 to 47.7% (22). Furthermore, data about the incidence of postoperative ARDS in patients undergoing hepatectomy is scarce. In addition, no predictors or prediction models for evaluating the possibility of developing ARDS in patients following hepatectomy were identified. Therefore, effective grades, predictive factors, or prediction models for early identifying the risk of developing ARDS in patients undergoing hepatectomy are extremely valuable.

Accordingly, by conducting a retrospective study in 1,032 patients undergoing hepatectomy between 2019 and 2020, we assessed potential predictors and constructed two prediction models to early predict the development of ARDS based on perioperative factors.

## Materials and methods

### Study design

This was a retrospective, single-center cohort study conducted at the Eastern Hepatobiliary Surgery Hospital, Shanghai, China. The study was approved by the Ethics Committee of the Eastern Hepatobiliary Surgery Hospital (no. EHBHKY2021-K-011) and adhered to Helsinki Declaration and the Strengthening the Reporting of Observational Studies in Epidemiology (STROBE) criteria. Patients who signed the informed consent form and authorized permission for future research use of their medical records were included.

### Participants

Patients were included between 1 January 2019, and 31 December 2020, based on the following criteria: (1) over the age of 18, (2) American Society of Anesthesiologists (ASA) Status I–III, (3) Child-Pugh class A or B, and (4) elective hepatectomy for liver cancer treatment, including hepatocellular carcinoma (HCC) and intrahepatic bile duct cancer. Patients who denied permission to utilize their health information for research purposes, as well as those with severe organ failure prior to surgery or a history of ALI or ARDS, were excluded from this study. It should be noted that liver failure was defined according to guidelines (23), heart failure as having a left ventricular ejection fraction less than 35%, respiratory failure as having an arterial oxygen partial pressure less than 60 mmHg, and renal failure as having a serum creatinine level greater than 442 μmol/L.

### Variables and outcomes

The preoperative clinical characteristics of the patients were recorded. In addition, intraoperative and postoperative factors such as tumor-related data, surgical data, fluid transfusion, and postoperative complications were collected. Tumor size is expressed as the maximum tumor diameter or the sum of maximum diameters when the tumor number exceeds one. Furthermore, laboratory results of liver function, renal function, inflammatory, and other biomarkers were collected preoperatively and postoperatively within 24 h after surgery. All data were collected from the digital medical system or paper medical records by two trained researchers, checked, and entered into Excel or the EpiData system.

The outcome of this study was the development of ARDS within the first 7 days after hepatectomy. ARDS was evaluated and diagnosed by experienced ICU physicians according to the Berlin Definition (14). The diagnosis of ARDS could be listed as follow: (1) Within 1 week of a known clinical insult or new or worsening respiratory symptoms; (2) bilateral opacities of chest imaging which could not fully explained by effusions, lobar/lung collapse, or nodules; (3) respiratory failure which could not fully explained by cardiac failure or fluid overload based on the examination of electrocardiogram or echocardiogram; (4) hypoxemia which identified by the arterial blood gas analysis. (5) Comprehensive analysis based on the clinical symptom, the biochemical detection of blood and results of bacterial culture. Furthermore, ARDS prediction models were constructed using the aforementioned factors and tested in two cohorts.

### Establishment and validation of prediction models

Patients with HCC or intrahepatic bile duct cancer who underwent hepatectomy in 2019 or 2020 were included in the development and validation cohorts, respectively. For prediction model construction, a logistic regression model and a least absolute shrinkage and selection operator (LASSO) model were used. Potential predictors with *p*-values of less than 0.05 in the multivariable logistic regression were selected for model construction. The effectiveness of the prediction model was validated in the validation cohort. Following that, a LASSO model was used to screen the most significant factors that contributed to the development of ARDS in patients. The risk score was calculated using the same method as described above, and the model was also validated in the validation cohort.

Due to the lack of an external data set for model validation, a 10-fold cross-validation method was used in both models. Furthermore, the nomogram was developed to offer a reliable and quantifiable method for immediately confirming the ARDS probability in two prediction models.

### Statistical analysis

The statistical analyses were performed and graphics were designed using the R software, version 4.0.0^1^ and the SPSS statistics software, version 23.0 (IBM SPSS Inc., Armonk, NY, USA). Categorical variables were presented as numbers and percentages, while continuous variables were expressed as mean and standard deviation or median (25% interquartile range, 75% interquartile range) based on their normality. Continuous variables were compared using the Student’s *t*-test or Mann-Whitney *U* test. Categorical variables were compared using the Chi-squared χ^2^ test. The receiver operator characteristic (ROC) curve was plotted and the area under the curve (AUC) was calculated to assess the model discrimination. Furthermore, calibration curves with C-index were made in two cohorts to test the prediction model stability. The optimal risk score cutoff point was determined by Youden’s index. The multiple imputation method was used to fill in the missing data for characteristics included in the prediction models, and all missing proportions were less than 10%. All statistical tests were two-sided, with *p*-values less than 0.05 considered statistically significant.

## Results

### Cohort characterization

According to the inclusion criteria, 490 patients were included in the development cohort and 542 in the validation cohort (Table 1). The incidence rate of ARDS was 8.8% (43/490) in the development cohort and 5.7% (31/542) in the validation cohort. The overall incidence rate of ARDS in patients undergoing hepatectomy was 7.2% (74/1,032). Several characteristics, such as weight, viral hepatitis, portal vein tumor thrombus, operation time, and others, were significantly different between the two groups. The incidence rate of postoperative complications was higher in the development cohort (32.0%) than in the validation cohort (25.8%). In particular, the incidence rate of pulmonary or abdominal infections was higher in the development cohort (10.8%) than in the validation cohort (3.9%).

**TABLE 1:** Clinical characteristics of patients in the development cohort and validation cohort.

Characteristics	Development cohort (*n* = 490)	Validation cohort (*n* = 542)	*P*-value
**Preoperative**			
Gender (male/female)	395/95 (80.6%/19.4%)	427/115 (78.8%/21.2%)	0.466
Age (year)	56.6 (11.5)	57.1 (10.8)	0.519
Height (cm)*	167.6 (6.3)	167.1 (6.7)	0.251
Weight (kg)*	67.8 (10.2)	66.3 (9.8)	0.027
ASA stage			0.204
I and II	424 (86.5%)	483 (89.1%)	
III	66 (13.5%)	59 (10.9%)	
Child-Pugh stage (A/B)	469/21 (95.7%/4.3%)	514/28 (94.8%/5.2%)	0.507
TNM stage			0.562
I	281 (57.3%)	326 (60.1%)	
II	156 (31.8%)	156 (28.8%)	
III and IV	53 (10.8%)	60 (11.1%)	
Hypertension (yes/no)	112/378 (22.9%/77.1%)	126/416 (23.2%/76.8%)	0.882
Diabetes (yes/no)	59/431 (12.0%/88.0%)	69/473 (12.7%/87.3%)	0.737
Smoking (yes/no)	196/294 (40.0%/60.0%)	245/297 (45.2%/54.8%)	0.092
Alcohol drinking (yes/no)	156/334 (31.8%/68.2%)	168/374 (31.0%/69.0%)	0.771
Viral hepatitis^§^ (yes/no)	370/120 (75.5%/24.5%)	296/246 (54.6%/45.4%)	0.000
HBV-DNA < 50 IU/ml (yes/no)	244/246 (49.8%/50.2%)	297/245 (54.8%/45.2%)	0.108
Cirrhosis (yes/no)	254/236 (51.8%/48.2%)	268/274 (49.4%/50.6%)	0.443
PVTT (yes/no)	35/455 (7.1%/92.9%)	20/522 (3.7%/96.3%)	0.014
Portal hypertension (yes/no) ^§^	78/412 (15.9%/84.1%)	99/443 (18.3%/81.7%)	0.318
TACE before surgery (yes/no)*^§^	47/443 (9.6%/90.4%)	58/482 (10.7%/89.3%)	0.543
**Intraoperative**			
Open/laparoscopic*	460/30 (93.9%/6.1%)	491/49 (90.9%/9.1%)	0.075
Left/right/caudate/left + right lobe resection*	131/309/6/44	117/344/8/67	0.129
Tumor number (single/multiple)	418/72 (85.3%/14.7%)	442/100 (81.5%/18.5%)	0.106
Tumor size (cm) ^§^	5.6 (3.8)	5.7 (3.8)	0.773
Operation time (min)	188.8 (94.5)	172.6 (82.3)	0.003
Volume of bleeding (ml)	300 [200, 500]	200 [200, 300]	0.000
Plasma transfusion (ml)	0 [0, 0]	0 [0, 0]	0.428
RBC transfusion (ml)	0 [0, 0]	0 [0, 0]	0.333
Crystalloid fluid** (ml)	1,400 [1,000, 1,500]	1,000 [1,000, 1,500]	0.001
Colloidal fluid** (ml)	500 [500, 1,000]	500 [500, 1,000]	0.639
**Postoperative**			
Postoperative complications^§§^	157 (32.0%)	140 (25.8%)	0.028
ARDS	43 (8.8%)	31 (5.7%)	0.057
Mild	31 (6.3%)	22 (4.1%)	0.590
Moderate	8 (1.6%)	4 (0.7%)	
Severe	4 (0.8%)	5 (0.9%)	
Fever^§§^ (>38^°^C over 48 h)	36 (7.3%)	46 (8.5%)	0.499
Pain	29 (5.9%)	37 (6.8%)	0.552
Bleeding	1 (0.2%)	4 (0.7%)	0.377
Pulmonary/abdominal infection^§§^	53 (10.8%)	21 (3.9%)	0.000
Severe PONV^§§^	1 (0.2%)	3 (0.6%)	0.626

Variables are shown as “mean (SD),” “number (%),” or “median [25% quartile, 75% quartile].”

ASA, American Society of Anesthesiologists; TNM, clinicopathological stage; HBV, hepatitis B viral; PVTT, portal vein tumor thrombus; TACE, transcatheter arterial chemoembolization; RBC, red blood cell; ARDS, acute respiratory distress syndrome; PONV, post operative nausea and vomiting; SD, standard deviation.

*Factors with a single asterisk indicate patients with missing data.

**Crystalloid fluid means lactated Ringer’s solution and colloidal fluid means hydroxyethyl starch solution (Voluven).

^§^Viral hepatitis includes HBV and HCV infection. Portal hypertension is defined as gastroscopy revealing esophageal varices or blue earthworm-like changes, TACE before surgery is defined as patients receiving TACE before surgery within 3 months.

^§§^Patients with multiple postoperative complications were counted separately. The body temperature is defined as the armpit temperature. Pulmonary infection is identified by sputum bacterial cultures and abdominal infection is identified by ascitic fluid bacterial cultures. Severe PONV is defined as episodes of the expulsion of gastric contents that need antiemetic treatment.

In addition, the laboratory results of 14 critical liver, renal, inflammatory, and coagulation-related biomarkers were collected preoperatively and postoperatively for model construction (Table 2). The levels of several biomarkers, such as lactate dehydrogenase (LDH), serum albumin (ALB), C-reactive protein, and others, were significantly different between the two cohorts.

**TABLE 2:** Laboratory detections of patients in the development cohort and validation cohort.

Biomarkers	Development cohort (*n* = 490)	Validation cohort (*n* = 542)	*P*-value
Hepatic function biomarkers*			
APT ≤ 40 mAU/ml (yes/no)	162/328 (33.1%/66.9%)	164/378 (30.3%/69.7%)	0.333
TBIL			
Pre-operation	13.4 [9.5, 17.6]	13.5 [10.3, 18.2]	0.124
Post-operation	27.5 [18.7, 39.5]	28.9 [21.0, 41.3]	0.057
ALT			
Pre-operation	27.0 [19.0, 41.0]	26.0 [18.0, 40.0]	0.432
Post-operation	269.0 [148.0, 461.8]	244.0 [142.5, 461.8]	0.527
AST			
Pre-operation	28.0 [21.0, 38.3]	27.0 [20.0, 38.0]	0.579
Post-operation	223.0 [131.8, 431.5]	225.0 [137.0, 415.5]	0.602
LDH			
Pre-operation	172.0 [150.0, 203.5]	166.0 [146.0, 197.0]	0.030
Post-operation	362.0 [258.8, 523.5]	330.0 [248.0, 473.5]	0.021
ALB			
Pre-operation	41.2 (4.3)	41.8 (4.0)	0.032
Post-operation	39.7 (5.9)	41.5 (6.1)	0.000
**Renal function biomarkers**			
Cr			
Pre-operation	75.1 (16.5)	74.0 (15.4)	0.282
Post-operation	75.1 (33.8)	69.4 (18.3)	0.001
BUN			
Pre-operation	5.4 [4.3, 6.4]	5.2 [4.3, 6.1]	0.099
Post-operation	4.2 [3.3, 5.3]	3.9 [3.0, 4.9]	0.000
**Inflammatory biomarkers**			
CRP			
Pre-operation	2.5 [2.5, 5.0]	2.5 [2.5, 5.0]	0.017
Post-operation	25.7 [12.4, 53.6]	31.6 [17.8, 63.4]	0.000
WBC			
Pre-operation	5.4 (2.6)	5.4 (2.1)	0.572
Post-operation	13.8 (5.2)	13.4 (9.3)	0.472
N %			
Pre-operation	59.2 (9.8)	59.0 (10.9)	0.761
Post-operation	86.5 (6.3)	85.9 (7.9)	0.212
**Others**			
Hb			
Pre-operation	138.1 (20.2)	140.0 (17.8)	0.126
Post-operation	121.7 (23.7)	122.0 (20.6)	0.842
PLT			
Pre-operation	155.7 (66.4)	158.9 (71.2)	0.456
Post-operation	141.6 (64.2)	140.4 (60.3)	0.766
INR			
Pre-operation	1.0 [1.0, 1.1]	1.0 [1.0, 1.1]	0.002
Post-operation	1.2 [1.1, 1.3]	1.2 [1.1, 1.3]	0.000

Variables are shown as “mean (SD),” “number (%),” or “median [25% quartile, 75% quartile].”

APT, abnormal prothrombin; TBIL, total bilirubin; ALT, alanine transaminase; AST, aspartate aminotransferase; LDH, lactate dehydrogenase; ALB, serum albumin; Cr, creatinine; BUN, blood urea nitrogen; CRP, C-reactive protein; WBC, white blood cell; N %, neutrophil %; Hb, hemoglobin; PLT, platelet; INR, international normalized ratio.

*Laboratory detections within 24 h after surgery were acquired for post-operation time point.

### Model construction and validation based on logistic regression

First, a logistic regression model was used to construct the prediction model. It included 18 covariates with *p*-values of less than 0.05 in the univariable logistic analysis (Supplementary Table 1). Three critical predictors; operation time, postoperative aspartate aminotransferase (AST), and postoperative hemoglobin (Hb); were identified and integrated into the prediction model (Supplementary Table 2). The risk score was calculated using the following formula:


Risk⁢score=(0.006×o⁢p⁢e⁢r⁢a⁢t⁢i⁢o⁢n⁢t⁢i⁢m⁢e)

                              +(0.001×p⁢o⁢s⁢t⁢o⁢p⁢e⁢r⁢a⁢t⁢i⁢v⁢e⁢A⁢S⁢T)

                              +(-0.031×p⁢o⁢s⁢t⁢o⁢p⁢e⁢r⁢a⁢t⁢i⁢v⁢e⁢H⁢b)

**FIGURE 1:**
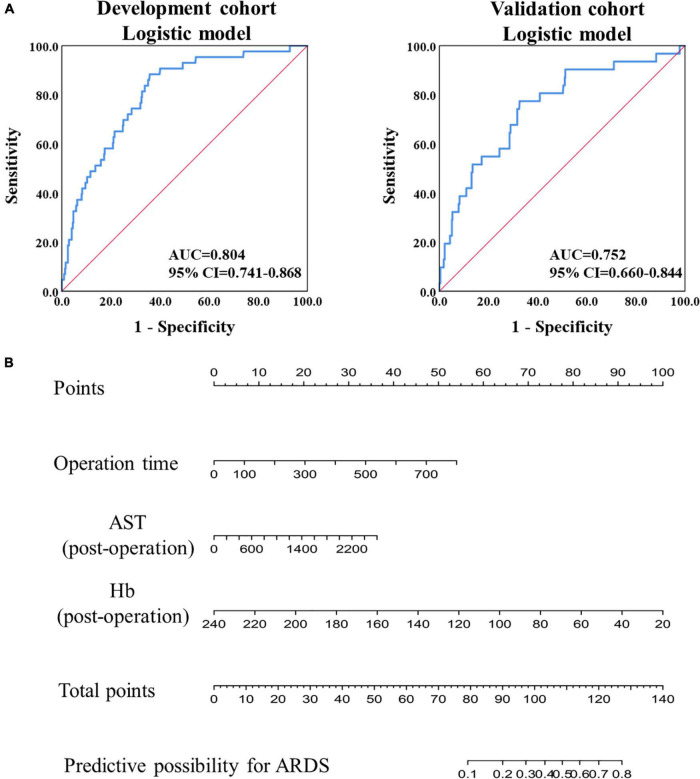
Model construction and validation based on logistic regression. **(A)** The ROC analysis of the prediction model in the development and validation cohorts. **(B)** The nomogram of prediction model based on the logistic regression in the development cohort.

The AUC was calculated using the ROC curve, and it was 0.804 (95% confidence interval (CI): 0.741–0.868) in the development cohort and 0.752 (95% CI: 0.660–0.844) in the validation cohort (Figure 1A). Furthermore, model validation using the 10-fold cross-validation method revealed similar discrimination with a mean C-index of 0.792 in the development cohort. Then, using raw data, a model was constructed, and a similar AUC was found in the two cohorts, with mean AUC values of 0.805 and 0.773 in the development and validation cohorts, respectively. Based on the logistic prediction model, a visual nomogram was developed to establish a reliable and quantitative method for assessing the probability of ARDS (Figure 1B).

### Model construction and validation based on LASSO regression

**FIGURE 2:**
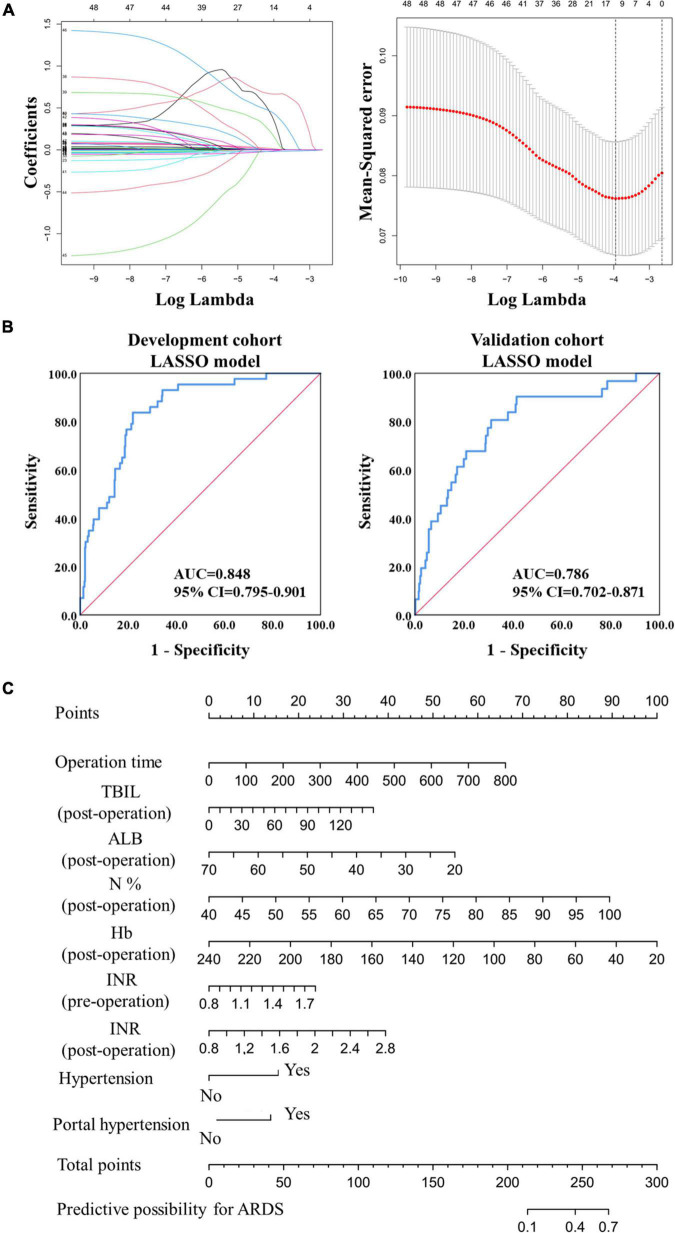
Model construction and validation based on LASSO regression. **(A)** The construction of prediction model based on the LASSO regression. **(B)** The ROC analysis of the prediction model in the development and validation cohorts. **(C)** The nomogram of the prediction model based on the LASSO regression in the development cohort.

A LASSO model was then used to construct another prediction model. Finally, 14 predictors were acquired, and nine predictors with coefficients greater than 0.001 were used in the final prediction model (Figure 2A). The risk score was calculated using the following formula:


R⁢i⁢s⁢k⁢s⁢c⁢o⁢r⁢e=(0.0033×o⁢p⁢e⁢r⁢a⁢t⁢i⁢o⁢n⁢t⁢i⁢m⁢e)

                              +(0.0048×p⁢o⁢s⁢t⁢o⁢p⁢e⁢r⁢a⁢t⁢i⁢v⁢e⁢T⁢B⁢I⁢L)

                              +(-0.0105×p⁢o⁢s⁢t⁢o⁢p⁢e⁢r⁢a⁢t⁢i⁢v⁢e⁢A⁢L⁢B)

                              +(0.01×p⁢o⁢s⁢t⁢o⁢p⁢e⁢r⁢a⁢t⁢i⁢v⁢e⁢N%)

                              +(-0.0187×p⁢o⁢s⁢t⁢o⁢p⁢e⁢r⁢a⁢t⁢i⁢v⁢e⁢H⁢b)

                              +(0.299×p⁢r⁢e⁢o⁢p⁢e⁢r⁢a⁢t⁢i⁢v⁢e⁢I⁢N⁢R)

                              +(0.664×p⁢o⁢s⁢t⁢o⁢p⁢e⁢r⁢a⁢t⁢i⁢v⁢e⁢I⁢N⁢R)

                              +[0.128×Hypertension (Yesrepresents1and

                              Norepresents 0)]+[0.418×portalhypertension

                              (Yesrepresents 1 andNorepresents 0)].

The AUC value reached 0.848 (95% CI: 0.795–0.901) in the development cohort and 0.786 (95% CI: 0.702–0.871) in the validation cohort (Figure 2B), exceeding the logistic prediction model’s discriminating power. In addition, model validation using the 10-fold cross-validation method showed similar discrimination, with a mean C-index of 0.808 in the development cohort. Furthermore, a visual nomogram was developed to help visualize the prediction model and directly calculate the probability of ARDS (Figure 2C).

**FIGURE 3:**
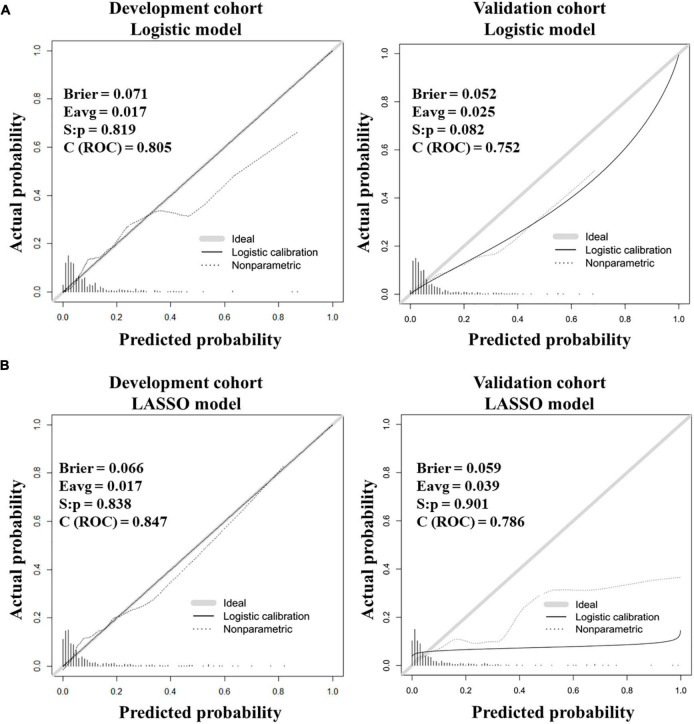
Calibration curves for testing the stability of prediction models in the development and validation cohorts. **(A)** Calibration curves for the logistic model. **(B)** Calibration curves for the LASSO model.

Furthermore, the calibration curves showed good fitting effects between predicted and observed outcomes for the logistic prediction model (Figure 3A) and the LASSO prediction model (Figure 3B) in two cohorts.

### Discriminative effects of prediction models for ARDS by grading risk scores

To better evaluate the discrimination of our prediction models and simplify models for clinical application, risk scores were calculated based on formulas and graded to three degrees: low-risk, medium-risk, and high risk. According to Table 3, patients’ risk scores were distributed consistently in the development and validation cohorts, regardless of whether the logistic or the LASSO model was used, suggesting good robustness of our models. We were able to effectively classify patients into different risk groups by dividing the risk score into three degrees. Based on our cohorts, the incidence of ARDS was approximately 2% in the low-risk group, while it significantly increased in the medium-risk and high-risk groups in both cohorts (Table 3).

**TABLE 3:** Incidences of ARDS classified by risk scores in the development cohort and validation cohort.

Degrees of risk score*	Incidences of ARDS	
	Development cohort (*n* = 490)	Validation cohort (*n* = 542)
**Logistic model:**		
Overall**	−2.42 [−3.06, −1.68]	−2.5 [−3.14, −1.81]
≤−2.15 (low-risk)	6/294 (2.0%)	8/357 (2.2%)
−2.15 to −1.15 (medium-risk)	18/134 (13.4%)	11/118 (9.3%)
≥−1.15 (high-risk)	19/62 (30.6%)	12/67 (17.9%)
**LASSO model:**		
Overall**	0.22 [−0.23, 0.73]	0.19 [−0.23, 0.74]
≤0.40 (low-risk)	4/299 (1.3%)	6/339 (1.8%)
0.40 to 1.22 (medium-risk)	20/127 (15.7%)	12/139 (8.6%)
≥1.22 (high-risk)	19/64 (29.7%)	13/64 (20.3%)

*The cutoff of risk scores was determined by the ROC curves in the development cohort. The lower bound was defined as the maximum of risk score when the sensitivity ≥ 90%, and the upper bound was defined as the minimum of risk score when the specificity ≥ 90%.

**The distribution of risk score was expressed as “median [25% quartile, 75% quartile].”

In the development cohort using the optimal cutoff, the logistic model exhibited a sensitivity of 88.4% and a specificity of 64.2% (Supplementary Table 3), while the LASSO model had a sensitivity of 83.7% and a specificity of 78.1% (Supplementary Table 3), suggesting good predictive effects of our prediction models for ARDS.

### Subgroup analysis of two prediction models in the development cohort

To verify the sensitivity of two models for predicting ARDS, subgroup analysis was performed in the development cohort. Table 4 shows that both prediction models had sustained predictive effects for ARDS in different subgroups. The AUC value reached 0.887 in the LASSO model in patients less than 60 years old. Furthermore, in the subgroup of patients with no hypertension, both logistic and LASSO models had superior predictive effects, with AUC of 0.858 and 0.892, respectively.

**TABLE 4:** Subgroup analysis of two prediction models for predicting ARDS in the development cohort.

Subgroups	Logistic model		LASSO model	
	AUC	95% CI	AUC	95% CI
**Gender**				
Male	0.806	0.729–0.883	0.843	0.781–0.906
Female	0.774	0.653–0.895	0.867	0.776–0.958
**Age**				
<60 years	0.835	0.765–0.905	0.887	0.833–0.941
≥60 years	0.776	0.674–0.879	0.809	0.722–0.897
**TNM**				
I	0.799	0.684–0.913	0.854	0.767–0.941
II–IV	0.794	0.722–0.865	0.827	0.758–0.896
**Smoking**				
Yes	0.787	0.682–0.892	0.820	0.716–0.924
No	0.817	0.737–0.897	0.863	0.804–0.922
**Alcohol drinking**				
Yes	0.816	0.700–0.932	0.810	0.686–0.933
No	0.803	0.726–0.879	0.865	0.811–0.919
**Cirrhosis**				
Yes	0.828	0.732–0.924	0.872	0.801–0.942
No	0.784	0.701–0.867	0.828	0.750–0.906
**Hypertension**				
Yes	0.683	0.538–0.828	0.749	0.609–0.888
No	0.858	0.800–0.916	0.892	0.850–0.934

AUC, Area under the curve; CI, confidence interval; TNM, clinicopathological stage.

## Discussion

Acute respiratory distress syndrome is one of the most devastating postoperative complications and significantly increased mortality. Early prediction and treatment of ARDS are critical for improving patients’ prognosis in clinical practice. To the best of our knowledge, our study was the first to report the incidence of ARDS in patients undergoing hepatectomy for liver cancer treatment, which was approximately 7.2%. In addition, we constructed two effective prediction models by integrating 48 perioperative covariates using logistic and LASSO regression. Both prediction models have good predictive effects, and the AUC exceeds 0.8 in the development cohort. Moreover, a number of major risk factors for postoperative ARDS have been identified. For instance, patients with a longer operation time, higher postoperative levels of AST, TBIL, and N %, and lower postoperative Hb and ALB levels are more likely to develop ARDS postoperatively. Furthermore, the risk of developing ARDS is effectively assessed by grading risk scores to three degrees. It enables clinicians to immediately identify patients at risk of ARDS.

Previously, a single-center retrospective study reported an acute lung injury (ALI) prediction model called the Lung Injury Prediction Score (LIPS) (24), and they developed a tool for evaluating the risk of ALI and ARDS in a population-based sample, which was validated in a multicenter study (16). Other studies, which focused on surgical lung injury, developed two scoring systems termed SLIP and SLIP-2 to predict the risk of early postoperative ALI and ARDS in patients undergoing elective surgery (17, 19). These studies supplied effective scoring systems for clinical practice.

Several predictors and prediction models for predicting the incidence of ARDS in patients with specific diseases or injuries have been reported (18, 25, 26). For instance, Majid et al. constructed a 3-variable model comprising total body surface area percent, inhalation injury, and von Willebrand factor-A2 to predict ARDS in patients with burn injuries, with an AUC of 0.90. In addition, Ning et al. developed a logistic model to predict the in-hospital incidence of ARDS in patients with acute pancreatitis (26). In addition, other studies reported the risk factors of ARDS in patients undergoing major surgery or extracorporeal membrane oxygenation (16, 18, 27–29). For instance, James et al. analyzed preoperative and intraoperative predictors of postoperative ARDS in a general surgical population (30). However, studies focusing on the incidence and early prediction models of ARDS in hepatobiliary surgery are scarce.

The logistic prediction model identified operation time, postoperative AST, and postoperative Hb as the most crucial predictors of ARDS. The operation duration reflects the complexity of the operation, and a longer operation duration usually means more trauma for the patients, as mentioned in another study on ARDS prediction (18). Furthermore, several studies found associations between liver function biomarkers (TBIL, AST, ALB, and international normalized ratio) and ARDS progression (31–33). Hypertension and portal hypertension also contributed to the development of ARDS based on the LASSO model (34, 35). It is possible to explain how portal hypertension can lead to ARDS as it is mainly caused by various forms of cirrhosis, which is closely associated with liver function and may aggravate ascites formation and hypoproteinemia. These predictors could be new biomarkers or targets for ARDS.

According to relevant studies, the incidence rate of ARDS following cardiac surgery ranges from 0.4 to 8.1% (17, 28, 36). Daryl et al. reported a 2.6% (113/4,366) incidence of ALI and ARDS in patients undergoing high-risk surgery, and 3.3% and 22% for patients undergoing spine surgery and high-risk vascular surgery, respectively (17, 19). In our study, the incidence of ARDS in patients undergoing hepatectomy was 7.2% (74/1,032), indicating a higher incidence rate of ARDS than in other types of surgery. Therefore, more attention should be paid to early prediction and prevention of ARDS development in patients undergoing hepatectomy.

In our study, two alternative prediction models were constructed, each with its own set of advantages. First, only three predictors were included in the logistic prediction model, making it easier and faster for clinicians to calculate risk scores and identify patients at risk for ARDS. Second, according to the AUC, the LASSO model with nine predictors outperforms the logistic model in predicting ARDS, with an AUC of 0.848 in the development cohort. However, predicting ARDS is more difficult for clinicians and requires more data from patients. Nevertheless, because only preoperative and postoperative (within 24 h after surgery) factors were included in the models, both models made it possible to early predict postoperative ARDS, saving time for pre-treatment of patients at high risk of ARDS.

There are some limitations to this study. First, due to the retrospective design of this study, there is unavoidably potential bias and confounding. Secondly, this is a single-center cohort, and prediction models are only validated using internal data. To compensate for this shortcoming, a 10-fold cross-validation method was employed to validate the robustness and accuracy of models simultaneously. Further studies are warranted to validate the results in more centers.

## Conclusion

In conclusion, we reported the incidence of ARDS in hepatobiliary surgery and developed two simple and practical prediction models for predicting postoperative ARDS in patients undergoing hepatectomy. These tools may improve clinicians’ ability to early estimate the risk of postoperative ARDS and timely prevent its emergence.

## Data availability statement

The original contributions presented in this study are included in the article/Supplementary material, further inquiries can be directed to the corresponding authors.

## Ethics statement

The studies involving human participants were reviewed and approved by the Ethics Committee of the Eastern Hepatobiliary Surgery Hospital. The patients/participants provided their written informed consent to participate in this study.

## Author contributions

XW, YL, and FW: study concept and design. XW, YL, HZ, and RZ: acquisition, analysis, and interpretation of data. XW and YL: statistical analysis, software, and drafting of the manuscript. FW and WY: critical revision of the manuscript. All authors contributed to the article and approved the submitted version.

## Footnotes

1
https://www.r-project.org/

## Supplementary material

Supplementary Table 1 Univariable logistic regression analyses for ARDS in the development cohort.

Supplementary Table 2 Multivariable logistic regression analyses for ARDS in the development cohort.

Supplementary Table 3 Performance of two prediction models in the development and validation cohorts.
