# Supplementary material for: Novel models for early prediction and prevention of acute respiratory distress syndrome in patients following hepatectomy: A clinical translational study based on 1,032 patients
Source: Front Med (Lausanne). 2023 Jan 9;9:1025764. doi: 10.3389/fmed.2022.1025764 (PMC9868423; doi:10.3389/fmed.2022.1025764)
Supplement: Supplementary Table 1 — Univariable logistic regression analyses for ARDS in the development cohort. [file Table_1.docx]

Supplementary Table 1. Univariable logistic regression analyses for ARDS in the development cohort.

| Factors | OR | 95% CI | *P* value |
| --- | --- | --- | --- |
| Gender (male vs female) | 1.485 | 0.719-3.067 | 0.285 |
| Age | 1.026 | 0.997-1.056 | 0.078 |
| ASA score (Ⅲ vs Ⅰ/Ⅱ) | 1.533 | 0.678-3.467 | 0.305 |
| Child-Pugh (A vs B) | 1.787 | 0.505-6.330 | 0.368 |
| TNM stage (Ⅲ/Ⅳ vs Ⅱ vs Ⅰ) | 1.496 | 0.978-2.289 | 0.063 |
| Hypertension (Yes vs No) | 1.719 | 0.874-3.380 | 0.116 |
| Diabetes (Yes vs No) | 1.775 | 0.780-4.036 | 0.171 |
| Smoking (Yes vs No) | 0.787 | 0.409-1.516 | 0.474 |
| Alcohol Drinking (Yes vs No) | 0.815 | 0.406-1.632 | 0.563 |
| Viral hepatitis (Yes vs No) | 0.726 | 0.366-1.442 | 0.361 |
| HBV-DNA < 50IU/ml (Yes vs No) | 0.866 | 0.462-1.621 | 0.652 |
| Cirrhosis (Yes vs No) | 1.075 | 0.574-2.014 | 0.820 |
| PVTT (Yes vs No) | 0.612 | 0.142-2.643 | 0.511 |
| **Portal hypertension (Yes vs No)** | **3.265** | **1.652-6.454** | **0.001** |
| **Tumor size** | **1.082** | **1.009-1.160** | **0.026** |
| **Operation time** | **1.006** | **1.004-1.009** | **0.000** |
| **Volume of bleeding** | **1.001** | **1.001-1.002** | **0.001** |
| **Plasma transfusion** | **1.002** | **1.001-1.003** | **0.002** |
| RBC transfusion | 1.001 | 1.000-1.001 | 0.065 |
| **Crystalloid fluid** | **1.001** | **1.000-1.001** | **0.020** |
| **Colloidal fluid** | **1.001** | **1.000-1.002** | **0.002** |
| APT ≤ 40 mAU/ml (Yes vs No) | 0.766 | 0.383-1.536 | 0.453 |
| TBIL (pre-operation) | 1.014 | 0.996-1.032 | 0.118 |
| **TBIL (post-operation)** | **1.024** | **1.011-1.036** | **0.000** |
| ALT (pre-operation) | 1.000 | 0.991-1.010 | 0.931 |
| ALT (post-operation) | 1.000 | 0.999-1.001 | 0.951 |
| AST (pre-operation) | 1.004 | 0.998-1.010 | 0.211 |
| **AST (post-operation)** | **1.001** | **1.000-1.002** | **0.005** |
| LDH (pre-operation) | 1.001 | 0.998-1.004 | 0.484 |
| LDH (post-operation) | 1.001 | 1.000-1.002 | 0.086 |
| **ALB (pre-operation)** | **0.913** | **0.849-0.982** | **0.014** |
| **ALB (post-operation)** | **0.936** | **0.882-0.994** | **0.030** |
| Cr (pre-operation) | 0.991 | 0.971-1.011 | 0.373 |
| **Cr (post-operation)** | **1.009** | **1.002-1.015** | **0.007** |
| BUN (pre-operation) | 0.960 | 0.807-1.141 | 0.642 |
| BUN (post-operation) | 1.006 | 0.977-1.036 | 0.682 |
| CRP (pre-operation) | 1.003 | 0.985-1.021 | 0.764 |
| CRP (post-operation) | 1.002 | 0.995-1.010 | 0.542 |
| WBC (pre-operation) | 0.912 | 0.785-1.059 | 0.227 |
| WBC (post-operation) | 1.026 | 0.969-1.087 | 0.377 |
| N % (pre-operation) | 1.022 | 0.989-1.055 | 0.193 |
| N % (post-operation) | 1.067 | 0.993-1.147 | 0.075 |
| **Hb (pre-operation)** | **0.981** | **0.968-0.994** | **0.005** |
| **Hb (post-operation)** | **0.962** | **0.949-0.976** | **0.000** |
| **PLT (pre-operation)** | **0.994** | **0.989-0.999** | **0.030** |
| **PLT (post-operation)** | **0.994** | **0.988-0.999** | **0.032** |
| **INR (pre-operation)** | **50.234** | **3.556-709.696** | **0.004** |
| **INR (post-operation)** | **13.539** | **3.740-49.007** | **0.000** |

ASA, American Society of Anesthesiologists; TNM, Clinicopathological stage; HBV, hepatitis B viral; PVTT, portal vein tumor thrombus; TACE, transcatheter arterial chemoembolization; RBC, red blood cell; APT, abnormal prothrombin; TBIL, total bilirubin; ALT, alanine transaminase; AST, aspartate aminotransferase; LDH, lactate dehydrogenase; ALB, serum albumin; Cr, creatinine; BUN, blood urea nitrogen; CRP, C-reactive protein; WBC, white blood cell; N %, neutrophil %; Hb, hemoglobin; PLT, platelet; INR, International Normalized Ratio; OR, odds ratios; CI, confidence interval.
