# Supplementary material for: Novel models for early prediction and prevention of acute respiratory distress syndrome in patients following hepatectomy: A clinical translational study based on 1,032 patients
Source: Front Med (Lausanne). 2023 Jan 9;9:1025764. doi: 10.3389/fmed.2022.1025764 (PMC9868423; doi:10.3389/fmed.2022.1025764)
Supplement: Supplementary Table 2 — Multivariable logistic regression analyses for ARDS in the development cohort. [file Table_2.docx]

Supplementary Table 2. Multivariable logistic regression analyses for ARDS in the development cohort.

| Factors | B | OR | 95% CI | *P* value |
| --- | --- | --- | --- | --- |
| Operation time | 0.006 | 1.006 | 1.002-1.009 | 0.001 |
| AST (post-operation) | 0.001 | 1.001 | 1.000-1.002 | 0.015 |
| Hb (post-operation) | -0.031 | 0.969 | 0.949-0.990 | 0.003 |

AST, aspartate aminotransferase; Hb, hemoglobin; OR, odds ratios; CI, confidence interval. B represents the regression coefficient.
