# Supplementary material for: Novel models for early prediction and prevention of acute respiratory distress syndrome in patients following hepatectomy: A clinical translational study based on 1,032 patients
Source: Front Med (Lausanne). 2023 Jan 9;9:1025764. doi: 10.3389/fmed.2022.1025764 (PMC9868423; doi:10.3389/fmed.2022.1025764)
Supplement: Supplementary Table 3 — Performance of two prediction models in the development and validation cohorts. [file Table_3.docx]

Supplementary Table 3. Performance of two prediction models in the development cohort and validation cohort.

| Performance measure* | Development cohort | | Validation cohort | |
| --- | --- | --- | --- | --- |
| Logistic model: ≥-2.15 |  |  | |  |
| Sensitivity % | 88.4 | 74.2 | |  |
| Specificity % | 64.2 | 68.3 | |  |
| Positive likelihood ratio | 2.47 | 2.34 | |  |
| Negative likelihood ratio | 0.18 | 0.38 | |  |
|  |  |  | |  |
| LASSO model: ≥0.67 |  |  | |  |
| Sensitivity % | 83.7 | 68.0 | |  |
| Specificity % | 78.1 | 75.0 | |  |
| Positive likelihood ratio | 3.82 | 2.72 | |  |
| Negative likelihood ratio | 0.21 | 0.43 | |  |

*Optimal cutoff point determined by Youden index was used to show the performance of two prediction models.
